# When to stop propranolol for infantile hemangioma

**DOI:** 10.1038/srep43292

**Published:** 2017-02-22

**Authors:** Lei Chang, Yifei Gu, Zhang Yu, Hanru Ying, Yajing Qiu, Gang Ma, Hui Chen, Yunbo Jin, Xiaoxi Lin

**Affiliations:** 1 From the Department of Plastic and Reconstructive Surgery, Shanghai Ninth People’s Hospital, Shanghai Jiao Tong University, School of Medicine, Shanghai, China

## Abstract

There is no definitive conclusion regarding the optimal timing for terminating propranolol treatment for infantile hemangioma (IH). A total of 149 patients who underwent detailed color Doppler ultrasound examination were included in this study. The characteristics and propranolol treatment of all patients were summarized and analyzed. Patients were divided into two groups according to the lesion regression rate. Among the 149 patients, 38 were assigned to the complete regression group, and 111 were assigned to the partial regression group. The age at which propranolol treatment started, duration of follow-up after treatment discontinuation and rate of adverse events were not significantly different between the two groups. The duration of oral propranolol treatment was shorter in the complete regression group. The age at which propranolol was terminated was younger in the complete regression group, and this group had a lower recurrence rate. Propranolol is safe and effective for the treatment of IHs that require intervention, but it should be stopped at an appropriate time, which is determined primarily by the lesion regression rate after propranolol treatment. Ultrasound is helpful in determining when to stop propranolol for IH.

With an occurrence rate of 4–10%^1^, infantile hemangioma (IH) is the most common benign vascular tumor. Propranolol, a nonselective beta-blocker with membrane-stabilizing activity, has become the first-line treatment for severe IHs^2^. Although some papers have described detailed therapeutic protocols, including the optimal timing for initiating treatment, pretreatment preparation, dosage, frequency of visits, and treatment duration, there is still debate surrounding the optimal timing for terminating propranolol treatment. In this study, we retrospectively examined 149 patients and determined the appropriate time to stop propranolol treatment for IHs.

## Results

**Figure 1:**
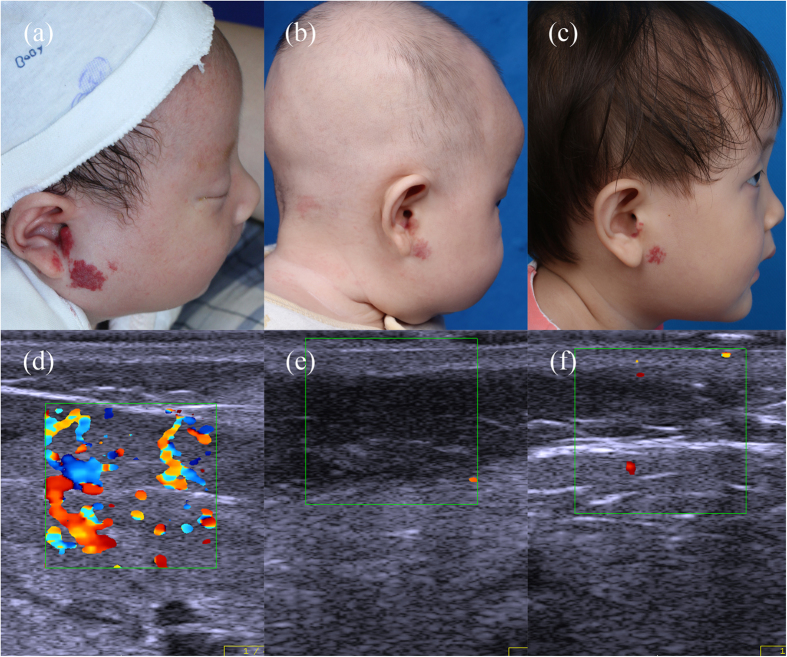
A typical complete regression case. (**a**) Before treatment, a 6-week-old girl presented with a mixed hemangioma in her right parotid region. (**b**) After 3 months of propranolol treatment, no obvious lesion remained. Propranolol was stopped gradually over 2 weeks. (**c**) The lesion was stable 23 months after propranolol was stopped. (**d,e,f**) Corresponding color Doppler ultrasound images of (**a,b** and **c**).

**Figure 2:**
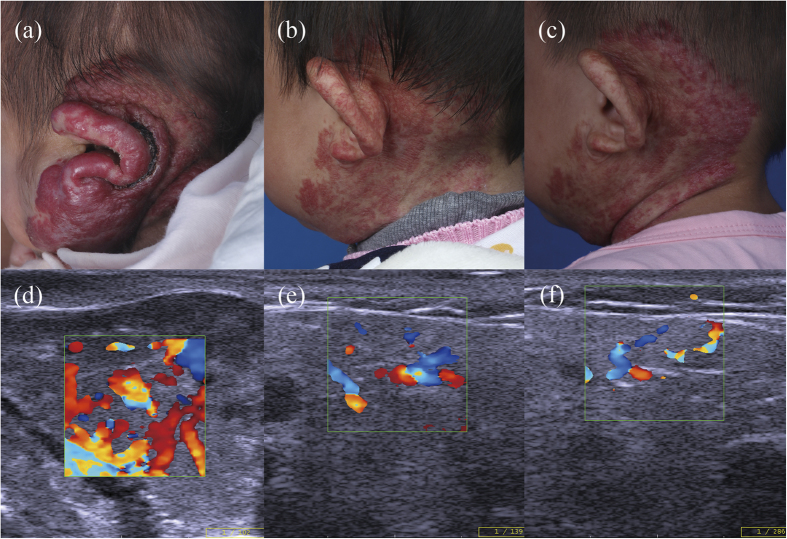
A typical case involving partial regression. (**a**) Before treatment, a 3-month-old girl presented with a large mixed hemangioma in her left parotid region. (**b**) After 15 months of propranolol treatment, maximal reduction was achieved for more than three months. Propranolol was stopped gradually over 4 weeks. (**c**) Six months after propranolol was stopped, the residual lesion remained stable. (**d,e,f**) Corresponding color Doppler ultrasound images of (**a,b** and **c**).

**Figure 3:**
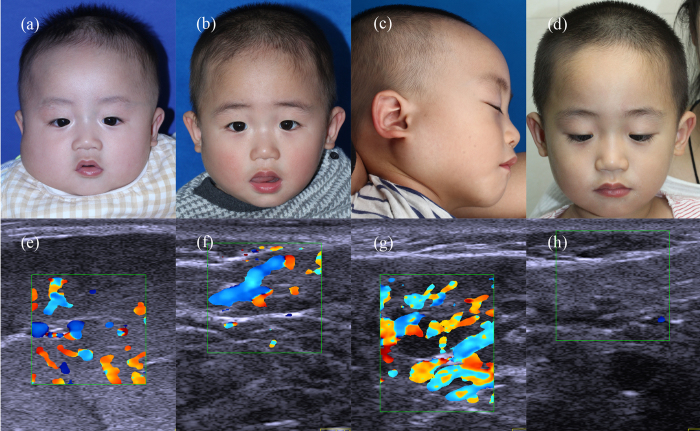
Another typical case involving partial regression. (**a**) Before treatment, a 4-month-old boy presented with a deep hemangioma in his right parotid region. (**b**) After 11 months of propranolol treatment, maximal reduction was achieved for more than three months. Propranolol was stopped gradually over 4 weeks. (**c**) One month later, progressive regrowth of the deep component of the hemangioma was observed. (**d**). No recurrence was observed 6 months after a second course of propranolol treatment for 7 months. (**e,f,g,h**) Corresponding color Doppler ultrasound images of (**a,b,c** and **d**).

According to the color Doppler ultrasound results at propranolol termination, patients (n = 149) were divided into two groups: the complete regression group (≥90% regression; n = 38) and the partial regression group (<90% regression; n = 111). In the complete regression group, the regression rate exceeded 90% compared with the pretreatment condition, or no obvious lesions remained (Fig. 1). By contrast, patients with less than 90% regression were included in the partial regression group (Figs 2 and 3). Table 1 summarizes the characteristics of the patients in the two groups, which had similarities. The use of propranolol in all patients is summarized in Table 2. The age at which propranolol treatment started, duration of follow-up after treatment discontinuation and rate of adverse events were not significantly different between the two groups. The duration of oral propranolol treatment was shorter in the complete regression group. The age at which propranolol was terminated was younger in the complete regression group, and this group had a lower recurrence rate. Compared with the pretreatment condition, the mean lesion depth and vessel density significantly decreased during the treatment course in both groups. The complete regression group showed better and more rapid regression than the partial regression group.

## Discussion

The proliferative phase of IH usually occurs during the first year of life. IH usually manifests during the first or second week of life and not later than 12 weeks of age. The majority of growth occurs before 12 weeks, after which growth velocity decreases and usually ceases between 4 and 6 months. Deep IHs appear somewhat later and grow somewhat longer than their superficial counterparts (on average, approximately 1 month more)^3^,^4^,^5^. Large, deeply penetrating lesions (particularly in the parotid area) can grow until the patient is 2 years of age^6^ but subsequently partially or completely disappear in 30% of patients by 3 years of age, 20% of patients by 5 years, and 30–40% of patients by 9 years; however, these lesions remain unchanged in 10–20% of patients^7^. Finn discovered that 81% of 159 patients in whom regression occurred before 6 years of age achieved a “perfect” cosmetic effect^8^. Thus, many physicians emphasize an approach of careful observation but not active treatment. These hemangiomas are usually not life threatening or function impairing. With advances in modern technology, active treatments not only exert definite therapeutic effects but also minimize the psychosocial distress caused by the lesions^9^. Propranolol has been the first-line treatment for severe IHs since 2008^2^. Given that IHs can involute spontaneously, the main aim of propranolol treatment is to control their growth or eliminate their impairment of vital organs. Most physicians suggest that propranolol should be used at least throughout the patient’s first year of life, which covers the entire proliferative phase.

Due to its potential side effects, particularly in the central nervous system^10^,^11^, propranolol use should continue until IHs are completely involuted in only rare circumstances. Nevertheless, terminating propranolol too early may lead to recurrence. Therefore, it is vital to determine the appropriate time at which to stop propranolol treatment for IHs, thereby decreasing its potential side effects and simultaneously ensuring curative effects.

Early termination of propranolol may relate to recurrence, but there is not yet a clear definition of recurrence. In the present study, IH recurrence was defined as obvious regrowth of a primary lesion and abundant blood flow as detected by color Doppler ultrasound. Because no further treatment was needed, patients with lesions with slight regrowth and without persistent growth were excluded. As most recurrent IHs require a long treatment course, they inevitably impose an intense psychological and economic burden on the patients’ families. Generally, there is a 10–30% recurrence rate after terminating propranolol treatment^12^,^13^. In our previous prospective study^14^ with a large sample (679 cases), the recurrence rate was 13.5% (92/679), and the mean age at propranolol termination was 11.1 months (range: 6–20 months). Szychta^15^ discontinued propranolol treatment after the end of the proliferative phase in children aged 12.3 months on average. Tan^16^ and Al^17^ suggested that propranolol should not be stopped until the lesion involutes or the child reaches 12 months of age. Al reported a rebound rate of 7% when propranolol was terminated at 1 year of age. In contrast, Holmes^18^ reported that treatment cessation at the earlier age of 6.5 months (range: 4 to 11.7 months) resulted in rebound growth in 24% of cases. However, Szychta^15^ found that most patients who experienced rebound growth were treated early, and surprisingly, propranolol was stopped relatively late at a mean age of 12.58 ± 3.51 months. Saqi^13^ reported that neither age at treatment onset nor treatment duration was a significant factor in IH relapse. Talaat^19^ reported that the optimal propranolol treatment duration must at least cover the entire IH proliferative phase and may last until the age of 12 months, especially for patients with subcutaneous components. After conducting a survey on the clinical use of propranolol for IHs in mainland China, Chen^20^ stated that the general rule was to continue therapy for at least the entire proliferative phase to mitigate the risk of recurrence. Because each IH responds differently to propranolol, patients in this study were divided into the complete regression and partial regression groups. The results showed that the partial regression group had a longer treatment course but a higher recurrence rate. In our experience, the point at which propranolol was terminated was mainly related to the patient’s sensitivity to propranolol, rather than the patient’s age or the length of the treatment course. Recurrence after terminating propranolol is most closely related to the IH regression rate at the time of termination. In our previous prospective study, we determined that recurrent cases were more common among patients whose lesions were difficult to resolve in a subsequent treatment course.

Because few dose-response studies have been conducted, the optimal dose of propranolol remains undetermined. The standard dose in most studies reported to date is 2 mg/kg/day (range: 0.5 to 3.0 mg/kg/day)^21^. As demonstrated in the randomized controlled study, 3 mg/kg/day was superior to 1 mg/kg/day, although studies comparing 2 mg/kg/day and 3 mg/kg/day have not been reported^2^. Sharma^22^ used a relatively low dose of propranolol (1.5 mg/kg/day) and speculated that the longer treatment duration compared to that in other studies may have been related to the relatively low dose. In a recent meta-analysis, Liu^23^ noted that the preferred dose of propranolol was at least 2 mg/kg/day. Another study reported that Chinese subjects have at least a twofold greater sensitivity to the beta-blocking effects of propranolol than Caucasian subjects. The free fraction of propranolol in plasma was shown to be 45% higher in Chinese subjects than in American individuals^24^. Therefore, a moderate dose, which we have demonstrated is safe and effective for Chinese infants, was utilized in our study for safety reasons.

Accurate, objective ways to measure the growth, involution and treatment response of IHs are important. There is no standard method for measuring hemangioma size or quantifying changes in size. Typically, IHs are classified as superficial, deep or compound. In most cases, propranolol is used to control the growth of deep or compound IHs, and it is therefore difficult to assess its efficacy by clinical observation alone. Most authors use the subjective visual analog scale (VAS), a modified global assessment scale, or try to measure changes in the surface area, volume, or thickness of IHs^25^. The objectivity and, more importantly, the reproducibility and reliability of these methods remain questionable. Moreover, they are commonly used for superficial IHs. Ultrasound examination has many advantages over other methods in evaluating the clinical efficacy of treatment for IHs^26^. Ultrasound can accurately detect IHs, distinguish them from vascular malformations, measure their depth and width, and clearly reveal their relationships to surrounding tissue. In addition, ultrasound is sensitive to blood flow signals and distinguishes arterial from venous blood flow. Magnetic resonance imaging (MRI) and computerized tomography (CT) are the gold standards for IH diagnosis and monitoring^27^ but often require general anesthesia in young children and are expensive to repeat. Therefore, ultrasound imaging is appealing for use in this population as a rapid and noninvasive means of evaluating IH size and monitoring the response to propranolol therapy. Bingham stated that gray-scale color Doppler ultrasound parameters can potentially be used to determine when to stop propranolol therapy, as the benefits of continued empiric propranolol therapy may be minimal with a maximal reduction in lesion volume and vessel density^28^. Color Doppler ultrasound was employed in our study to help determine when to stop propranolol. The IH regression rate can be clearly monitored by ultrasound. Given that each patient responds differently to propranolol, an individualized approach should be adopted. Additionally, the most important issue is determining when to stop propranolol; the ideal time is when complete regression occurs. However, more IHs partially regress than completely regress after propranolol treatment. In cases of partial regression, we suggest that propranolol can be stopped when patients are older than one year of age and achieve maximal reduction for more than three months. However, even though patients with partial regression usually experience a longer treatment course compared to those with complete regression, they often have a higher recurrence rate. The challenge is to improve the regression rate of partially regressed IHs with future propranolol treatment courses. Other therapies may be more effective for these patients.

Chen^20^ reported that tapering and directly stopping propranolol were advised at 40 and 52%, respectively, of 31 hospitals in China, with the same result of no occurrences of rebound. However, more physicians suggest that propranolol administration should be gradually tapered to minimize the risks of rebound growth and a hyper-adrenergic withdrawal response^15^,^29^. In our experience, direct termination of propranolol usually leads to rapid regrowth of primary lesions, especially for patients with partial regression. Therefore, we recommend that patients with complete regression gradually stop treatment over a period of 2 weeks and that patients with partial regression carefully terminate treatment over 4 weeks.

Propranolol is safer than other treatments, and its side effects are usually minor^24^. In our study, the rate of side effects was not significantly different between the complete regression and partial regression groups. Propranolol appears to be safe for long-term use. Moyakine did not detect evidence of psychomotor developmental delay among infants with IH who were treated with propranolol^11^ and found no increased developmental risk or growth impairment at 4 years of age in patients with IH who were treated with propranolol for 6 months or longer^30^. However, more physicians are cautious about using propranolol. We suggest that propranolol be stopped at an appropriate time to avoid potential side effects under the precondition of retaining its efficacy. Overuse of propranolol should be avoided.

Recently, other beta-adrenergic blockers, such as atenolol, have been reported as treatments for IH^31^. They appear to be as effective as propranolol with fewer side effects. Given the small number of cases reported in the literature, conclusions cannot be reached at this time.

Deep component and superficial erythema remained in some IHs at the last follow-up. Additional therapies, such as topical drugs, laser or surgery, may be necessary in these cases after terminating propranolol treatment. Longer follow-ups are needed. Further investigation is required to determine the mechanisms underlying different outcomes between patients with complete regression and those with partial regression after propranolol treatment and to improve the regression rate associated with cases of partial regression.

## Conclusion

Propranolol is safe and effective for the treatment of IHs that require intervention. It should be stopped at an appropriate time, which is primarily based on the lesion regression rate after propranolol treatment. The ideal time to terminate propranolol is when complete regression is achieved. However, more patients experience partial regression, and their treatment course should be longer; furthermore, the recurrence rate is frequently higher among these patients. The use of color Doppler ultrasound creates an opportunity to critically and accurately monitor the course of IHs to help determine when to stop treatment.

## Methods

The study was conducted in accordance with the Declaration of Helsinki. The procedures were approved by the ethics committee of Shanghai Ninth People’s Hospital (201206). Informed consent was obtained from each subject’s parents prior to treatment. From January 2012 to February 2016, propranolol treatment was indicated for 835 IHs following an assessment by two independent plastic surgeons according to the inclusion and exclusion criteria^15^. Propranolol was administered to patients at a dose of 2 mg/kg/day (in two divided doses with an interval of 12 hours that was slowly increased over the first three days). Propranolol was gradually stopped over two to four weeks, either when the patient was older than 12 months and maximal reduction was achieved for more than three months or when no obvious lesions remained, as evidenced by color Doppler ultrasound^32^. A routine visit every month or every two months was required for at least six months after discontinuing therapy. A total of 149 patients who underwent a color Doppler ultrasound (MyLab Touch; Esaote SpA, Genoa, Italy) examination during the therapeutic process and finished the subsequent follow-up (6 to 25 months) were included. Clinical measurements of a sleeping child were obtained by one author to determine the deepest spot of each IH. The lesion depth and vessel density were used as parameters because they can effectively reflect the lesion regression rate and vessel activity^26^,^28^. These parameters were recorded and analyzed before treatment, one month after initiating treatment and at treatment termination. The effect of propranolol was mainly evaluated by comparing the color Doppler ultrasound results at treatment termination with those before propranolol treatment. Patients who had received previous therapies prior to propranolol treatment were excluded from the present study to simplify the outcome expectations. Moreover, patients with multiple IHs were excluded from the study to facilitate the comparison of outcomes across different patients. All statistical analyses were performed using SPSS version 17.0. Lesion depth and vessel density were analyzed using Student’s t test (two-tailed). The significance threshold was set at 0.05.

## Additional Information

**How to cite this article:** Chang, L. *et al*. When to stop propranolol for infantile hemangioma. *Sci. Rep.* **7**, 43292; doi: 10.1038/srep43292 (2017).

**Publisher's note:** Springer Nature remains neutral with regard to jurisdictional claims in published maps and institutional affiliations.

## Figures and Tables

**Table 1:** Characteristics of patients with complete and partial regression.

	Complete regression group	Partial regression group
Female: male ratio	27:11	81:30
Premature birth	3	12
Low birth weight (<2500 g)	2	11
Nascent lesion at birth	7	19
Ulceration	4	12
Depth (cm)	2.03 ± 0.34	2.11 ± 0.41
Type		
Deep	16	43
Mixed	22	68
Site		
Head & Neck	24	69
Trunk	9	31
Limbs	5	11

**Table 2:** Propranolol treatment.

	Complete regression group	Partial regression group
Time (months)		
Age at treatment initiation	3.1 ± 1.2	3.4 ± 1.3
Treatment duration	6.7 ± 2.3	12.5 ± 3.3
Age at end of treatment	9.8 ± 3.6	16.9 ± 4.1
Follow up duration	11.7 ± 3.8	13.1 ± 4.2
Depth of lesion (cm)		
Before treatment	2.03 ± 0.34	2.11 ± 0.41
After 1 month of treatment	0.81 ± 0.23	1.29 ± 0.27
At end of treatment	0.12 ± 0.07	0.38 ± 0.12
Vessel density (/cm^2^)		
Before treatment	21.1 ± 5.3	22.9 ± 5.8
After 1 month of treatment	10.8 ± 3.2	13.1 ± 3.3
At end of treatment	1.7 ± 0.8	4.1 ± 1.3
Rate of adverse events	18.4% (7/38)	18.9% (21/111)
Recurrence rate	5.3% (2/38)	26.9% (24/111)
